# Spinal fusion with motor evoked potential monitoring using remimazolam in Alström syndrome: A case report

**DOI:** 10.1097/MD.0000000000027990

**Published:** 2021-11-24

**Authors:** Ayako Arashiro, Hayato Shinzato, Kota Kamizato, Manabu Kakinohana

**Affiliations:** Department of Anesthesiology, Faculty of Medicine, University of the Ryukyus, 207 Uehara, Nishihara-cho, Okinawa, Japan.

**Keywords:** Alström syndrome, case report, general anesthesia

## Abstract

**Rationale:**

Alström syndrome is a rare genetic disorder characterized by obesity, diabetes mellitus, cardiomyopathy, and liver dysfunction. Further, scoliosis, a common symptom of Alström syndrome, often requires surgical intervention for functional impairments. Motor evoked potential (MEP) monitoring and other electrophysiological tests are essential when performing surgery for functional scoliosis. However, there are few reports on how to maintain general anesthesia in Alström syndrome. Here, we describe a patient with Alström syndrome who underwent surgery for scoliosis under general anesthesia with remimazolam and MEP monitoring.

**Patient concerns:**

A 17-year-old woman (height, 140 cm, weight, 64.5 kg) diagnosed with Alström syndrome was scheduled for a posterior spinal fusion for functional scoliosis. Other associated comorbidities of Alström syndrome present were dilated cardiomyopathy, type 2 diabetes mellitus, obesity (body mass index, 32.1 kg/m^2^), amblyopia (light perception), and hearing impairment (speech awareness threshold 50 dBHL in each ear).

**Diagnoses, interventions, and outcomes:**

Posterior spinal fusion was planned for functional scoliosis. While investigating the dilated cardiomyopathy, transthoracic echocardiography showed global wall hypokinesis, with 45% left ventricular ejection fraction. The left ventricle was dilated, with left ventricular end-diastolic and end-systolic diameters of 55 and 42 mm, respectively. This finding along with the hypertriglyceridemia associated with Alström syndrome led us to conclude that propofol should be avoided. Thus, we induced general anesthesia using remimazolam. MEP monitoring was performed, and the patient experienced no motor impairments during the surgery.

**Lessons:**

Myocardial and hepatic dysfunction determine the prognosis of patients with Alström syndrome. Thus, anesthesia that preserves liver function should be selected in such cases. In patients with hypertriglyceridemia, propofol should be avoided, and using remimazolam, an ultrashort-acting benzodiazepine, may be appropriate. In this case, reviewing the Patient State Index with SedLine allowed us to perform MEP monitoring uneventfully, and the posterior spinal fusion was completed without any motor impairment.

## 1 Introduction

Alström syndrome is a rare autosomal recessive disorder comprising morbid obesity, dilated cardiomyopathy, diabetes mellitus, and dyslipidemia. To the best of our knowledge, the number of case reports describing anesthesia for patients with Alström syndrome is limited. Here, we describe a patient with Alström syndrome who underwent surgery for scoliosis under general anesthesia with remimazolam and motor evoked potential (MEP) monitoring.

## 2 Case

A 17-year-old woman (height, 140 cm, weight, 64.5 kg) diagnosed with Alström syndrome was scheduled for a posterior spinal fusion for functional scoliosis. Other associated comorbidities of Alström syndrome present were dilated cardiomyopathy, type 2 diabetes mellitus, obesity (body mass index, 32.1 kg/m^2^), amblyopia (light perception), and hearing impairment (speech awareness threshold 50 dBHL in each ear).

While investigating the dilated cardiomyopathy, transthoracic echocardiography showed global wall hypokinesis, with 45% left ventricular ejection fraction. The left ventricle was dilated, with left ventricular end-diastolic and end-systolic diameters of 55 and 42 mm, respectively. Her serum liver function and lipid profile were mostly normal (reference standard ranges): alanine aminotransferase 67 U/L (7–23), aspartate aminotransferase 35 U/L (13–30), total cholesterol 5.09 mmol/L (3.67–6.41), and triglyceride 2.32 mmol/L (0.77–3.02). No coagulation abnormalities were observed.

Anesthetic induction was performed using 5% sevoflurane inhalation. Next, continuous intravenous infusion with remifentanil was started at 0.3 μg/kg/min and remimazolam at 0.5 mg/kg/h following the termination of sevoflurane. After administration of rocuronium 30 mg as a muscle relaxant, tracheal intubation was performed with McGrath MAC video laryngoscope (Medtronic, MN). Sevoflurane was not an option for maintenance. Patient State Index (Psi) monitoring by SedLine (Masimo Corp, CA) was used to determine the maintenance dose of remimazolam, and it was adjusted between 40 and 60.

MEP monitoring was performed during the procedure using Neuropack, MEB-2312 (Nihon Kohden, Tokyo, Japan), and it was set up as we usually use. In summary, transcranial screw electrodes were used as stimulation electrodes, and the stimulation sites were C3 and C4. The stimulation method used was biphasic quadruple stimulation, with supramaximal stimulation before the skin incision as the reference value for stimulus intensity. The recording sites were the gastrocnemius, quadriceps, tibialis anterior, and abductor pollicis brevis. All sites were monitored bilaterally.

Although the initial rate of remimazolam administration of 0.5 mg/kg/h varied up to 1.0 mg/kg/h, no decrease in the amplitude of MEP was observed during the procedure.

The procedure involved posterior fixation of T4-L4 in the supine position, and the operation lasted 7 hours 40 minutes. Intraoperative blood loss was 1425 mL; however, autologous blood transfusion (700 mL), red blood cell transfusion (560 mL), intraoperative cell salvage (260 mL), and fresh–frozen plasma transfusion (480 mL) were performed. The circulatory and respiratory dynamics were stable in the perioperative period.

After the surgical procedure, the patient was transferred to the intensive care unit, with extubation performed the next day. The patient's respiratory status remained stable thereafter. No obvious limb paralysis was observed. Postoperative rehabilitation was continued in the ward, and the patient was able to walk independently from the 6th day after surgery. The patient was then discharged on the 21st day after surgery, and at a follow-up visit 1 and 3 months later, was deemed to have made good recovery.

The patient and her parents provided written informed consent for the publication of this case report.

## 3 Discussion

Alström syndrome is an extremely rare inherited disorder, first described by Carl Henry in Sweden in 1959; since then, approximately 950 cases have been reported worldwide.^[1]^ The syndrome is an autosomal recessive inherited disease that is associated with mutations in the autosomal *ALMS* gene (2p13.1). Symptoms are characterized by obesity, dilated cardiomyopathy, type 2 diabetes mellitus (due to insulin resistance), dyslipidemia, visual and hearing abnormalities, and liver and kidney dysfunction. Mental retardation is usually absent, while scoliosis is a common feature.^[2,3]^

During spinal and spinal cord surgery, intraoperative MEP monitoring is often performed for neurological evaluation. Generally, it is well-known that inhaled anesthetics, such as sevoflurane and desflurane, potentially suppress MEP amplitude.^[4]^ Therefore, continuous administration of intravenous anesthetics, especially propofol, is recommended as a sedative for spine surgery under MEP monitoring.^[5,6]^ However, hypertriglyceridemia can occur with propofol use even in patients with normal liver functions.^[7]^ Because this patient showed hypertriglyceridemia associated with Alström syndrome, it was decided that remimazolam, instead of propofol, should be used during this spine surgery under MEP monitoring.

Remimazolam, a recently developed ultra-short-acting benzodiazepine sedative, is a novel drug that can easily achieve a stable blood concentration.^[8]^ Further, the infusion rate can directly define the blood concentration. Therefore, it was thought that adequate hypnotic level for MEP monitoring could be easily maintained using by remimazolam. The use of benzodiazepines is also acceptable for spine surgery under MEP monitoring.^[5,6]^ However, since a previous study reported that midazolam was capable of suppressing the MEP amplitude induced by transcranial stimulation, there was the worry that remimazolam also might suppress MEP amplitude leading to failure in the MEP monitoring.^[9]^ Contrastingly, a case report, detailing 2 cases, showed that remimazolam and remifentanil anesthesia, using a PSi monitor to assess the optimal dose, could provide successful MEP monitoring for spine surgery.^[10]^ In our case, MEP montoring during spine surgery could be also performed by administering remimazolam at 0.5 mg/kg/h to 1 mg/kg/h using an EEG monitor, PSi.

Clinical reports about anesthetic management for Alström syndrome are limited. It is not clear which anesthetic should be used. However, our report is an early one with remimazolam.

In conclusion, remimazolam may be useful in surgery requiring MEP monitoring for Alström syndrome and in other cases where propofol infusion should be avoided.

## Acknowledgments

We would like to thank Editage (www.editage.com) for English language editing.

## Author contributions

**Conceptualization:** Kota Kamizato, Manabu Kakinohana.

**Data curation:** Ayako Arashiro, Kota Kamizato.

**Supervision:** Hayato Shinzato, Manabu Kakinohana.

**Writing – original draft:** Ayako Arashiro, Kota Kamizato.

**Writing – review & editing:** Hayato Shinzato, Manabu Kakinohana.
